# Demoralization and well-being among self-employed individuals with cardiac disease: the role of intolerance of uncertainty

**DOI:** 10.3389/fpsyg.2024.1388032

**Published:** 2024-07-03

**Authors:** Wafaa Sowan, David Kissane

**Affiliations:** ^1^School of Social Work, University of Haifa, Haifa, Israel; ^2^Centre for Palliative Care, Department of Medicine, University of Melbourne, Melbourne, VIC, Australia

**Keywords:** demoralization, well-being, self-employed, cardiac disease, intolerance of uncertainty, work ability limitations

## Abstract

**Background:**

Individuals with cardiac disease (CD) who are self-employed may experience ability limitations and especially intensive challenges and uncertainties. These challenges may cause demoralization and impaired well-being.

**Objectives:**

To examine: (a) whether work ability limitations are related to demoralization and well-being among self-employed people with CD; (b) rates of demoralization; and (c) how demoralization and intolerance of uncertainty (IU) are associated with well-being.

**Methods:**

The study involved 120 self-employed individuals with CD. The PROCESS macro was used to analyze mediation and moderation processes.

**Results:**

The prevalence of demoralization syndrome was 37.4%. Work ability-limitations were associated with higher demoralization levels. Demoralization was associated with well-being only among participants with high IU. Further, demoralization mediated the relationship between work ability limitations and well-being only for individuals with high IU.

**Conclusion:**

Encountering limitations among self-employed was associated with demoralization and lower levels of well-being, especially among those with high IU. In addition, demoralization syndrome is prevalent among individuals with CD in general. Early recognition and treatment of demoralization as a treatable psychological syndrome are essential for preventing its degeneration into more complex forms. In addition to uncertainty related to health, it is important to pay special attention to other sources of uncertainty.

## Highlights

- *What is known on the subject:* Individuals with CD who experience uncertainty have greater psychological problems. In addition, personality traits can influence the relationship between uncertainty and psychological problems.
- *What the paper adds to existing knowledge:* Demoralization syndrome is prevalent among individuals with CD in general and those who experience additional sources of uncertainty such as self-employed people, who can face especially intensive challenges and uncertainties. These challenges are associated with demoralization symptoms and impaired well-being, especially among those with high IU.
- *What are the implications for practice:* Early recognition and treatment of demoralization as a treatable psychological syndrome are essential for preventing its degeneration into more complex forms.

## Cardiac disease and self-employed

Individuals with cardiac disease (CD) often experience repeated periods of uncertainty regarding their health. In addition to this major stressor, this population often faces uncertainties in other areas, such as financial stability and family responsibilities. This study focused on a subgroup of individuals with cardiac disease who are also self-employed. This subgroup has to deal with various challenges in addition to their medical condition (Timmis et al., 2020).

The British Heart Foundation estimates that approximately 260 million men and 290 million women suffer from CD across the world (Go et al., 2014). Studies also have shown that about 45% of people with CD are of working age and employed (Gupta et al., 2014). Moreover, these figures are likely to increase in light of advances in CD management coupled with an aging workforce and the trend to delay retirement.

Although medical care has recently advanced, those with CD still experience physical, psychological, and social challenges in their daily lives (Thornhill et al., 2008), which can also lead to reduced work capacity and increased substantial economic strain. CD patients who are self-employed were found to encounter diverse difficulties (Krittanawong et al., 2020). This situation can increase uncertainty among self-employed people, thereby generating additional difficulties in maintaining their business. Stable and satisfying employment for self-employed people with CD, on the other hand, was found to be associated with longevity, better health status, higher personal satisfaction, and positive life adjustment (Boot et al., 2015).

Self-employment is a highly demanding type of work that may increase the uncertainty encountered by this population (Schonfeld and Mazzola, 2015; Smedegaard et al., 2017) through other unique challenges. These obstacles can include financial management (e.g., low profit margin, rent, lack of funding, high customer acquisition costs), work-related stress, unstable working schedule (e.g., working on weekends, no time for annual physical or routine doctor visits), and operational problems (e.g., inability to control expenses or overexpansion; Smedegaard et al., 2017; Krittanawong et al., 2020).

Such situations of uncertainty and consistent challenges may provide fertile ground for the development of negative mental states such as demoralization, which refers to a state of lowered morale and poor coping when feeling trapped in a situation that results in hopelessness, helplessness, and a loss of value, meaning, and purpose in life (Bobevski et al., 2021). These challenging circumstances and demoralization can also trigger an intolerance of uncertainty (IU). This inability to tolerate the discomfort or anxiety that arises from uncertainty is considered the fuel for ongoing worries (Bottesi et al., 2020). However, the effects of demoralization and IU on the well-being of self-employed individuals with CD have not been studied. This study explored the relationships among demoralization, IU, and well-being among self-employed individuals with CD to understand factors related to well-being in this population, which experiences uncertainty related to both work and health conditions.

### Demoralization and CD

CD is a serious illness that threatens the integrity of the body and mind and challenges a person’s sense of mastery and control (Clarke et al., 2005; Behan, 2018). It forces patients to navigate an unsettling world of symptoms, crucial treatments, and adaptation to new medical conditions and routines. The inescapable challenge of coping with serious health conditions can escalate to an existential crisis featuring a loss of sense of agency or control (Clarke et al., 2005). When encountering uncertainty regarding whether the disease will be resolved, people may develop a subjective sense of incompetence (Clarke and Kissane, 2002). If heart disease is severe and hinders active coping attempts, the disease may redefine a person’s life by introducing physical limitations and pain. This may alter family and occupational functioning and introduce the frightening possibility of permanent impairment or death, often leading to demoralization (Clarke and Kissane, 2002; Behan, 2018).

Demoralization following CD has identifiable symptoms and physiological, psychological, and social implications that warrant attention (Rzeszut and Assael, 2020). Individuals who have CD and experience demoralization symptoms were found to display somatization, psychological suffering, disease denial, significantly lower quality of life, and poorer psychosocial functioning (Sirri et al., 2011; Tecuta et al., 2014). Researchers tested demoralization symptoms among people with different medical conditions and found that demoralization influences health behaviors such as treatment compliance, inhibits adaptive coping, and increases the risk of suicide, negative disease outcomes, and medical costs (Robinson et al., 2015; Griffith, 2017; Liao et al., 2017; Wu et al., 2019). In addition, several studies have shown that demoralization has a mediated effect between stress and mental health (Robinson et al., 2017; Hsiu-Ling et al., 2021). In a study conducted by Hsiu-Ling et al. (2021), demoralization was shown to completely mediate the effects of stress on sleep disturbances and psychological wellbeing.

### Demoralization and IU

Despite the correlation between stresses concerning health and psychological outcomes, this stress is not always associated with negative psychological outcomes. Stress theories suggest that individual differences moderate the relationship between stress and the negative psychological outcomes (Lazarus and Folkman, 1984). Based on the established literature that feeling of uncertainty induces anxiety and stress (Gu et al., 2020). Intolerance of uncertainty is a personal tendency, it can be defined as the dispositional fear of the unknown (Carleton, 2012), which is a sub-trait of anxiety (Taylor, 2022). IU can be considered as a sub-trait of anxiety, which is often found in association with distress, psychosomatic symptoms, and other clinical conditions (Taylor, 2022). This tendency leads to faulty assessments of threat, and reduced coping abilities when confronted with uncertain or unpredictable future events (McEvoy and Mahoney, 2013; Tull et al., 2020).

IU among self-employed individuals with CD can significantly affect their work performance and mental and emotional well-being. According to Robichaud and Dugas’s (2006) model of IU and demoralization, a negative situation (e.g., CD) may lead to various uncertainties and raise questions such as, “What if my limitations worsen?” These worries are often fueled by IU and associated with anxiety and emotional discomfort (e.g., irritability). Experiencing these repeated and ongoing worries typically leads to or exacerbates feelings of demoralization and exhaustion (Butler et al., 1991). Subsequently, demoralization and exhaustion may impair well-being. We hypothesized that the effect of demoralization on well-being will be moderated by trait-level IU. To the best of our knowledge, no studies have examined IU (or related constructs) in the context of self-employed people with CD.

### Study context

In 2023, Israel had an employment rate of 70.1% and a self-employment rate of 12.4%, which is similar to other developed countries (OECD, 2023). Israel has approximately 350,000 self-employed individuals and about 200,000 small businesses (1–4 employees); together, they make up about 550,000 self-employed and small business owners, representing 21% of the nation’s business output (Israel Central Bureau of Statistics, 2022). In accordance with various employment agreements and employee work periods, the right to sick leave for employees is approximately 90 days (Ministry of Labor, n.d.). Self-employed people, unless experiencing a work accident, are entitled to sick leave compensation under private insurance only, but many do not take advantage of it because it is too expensive. After 90 days, every citizen is entitled to a disability allowance based on the percentage of medical disability determined, their ability to work, or their income up to 60% of the average wage (National Insurance Institute, n.d.).

**Figure 1:**
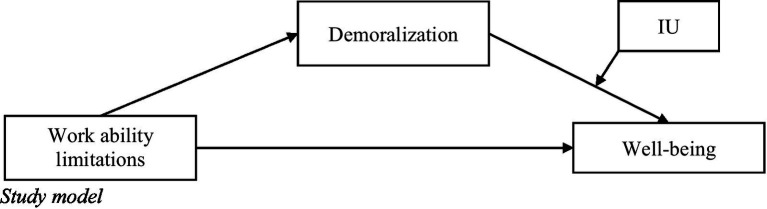
Study model.

Due to the lack of knowledge on factors that promote well-being among self-employed individuals with CD worldwide and in Israel, the present study aimed to examine: (a) how work ability limitations among CD patients are related to demoralization and well-being; (b) rates of demoralization among self-employed individuals with CD; and (c) how demoralization and IU are associated with well-being (see Figure 1 for study model).

## Method

### Participants and procedure

This study was approved by the ethics review board of the affiliated university [blinded for review]. The study featured 120 self-employed individuals with CD. The inclusion criteria was having suffered a heart attack in the past 2 years (experienced CD related symptoms or received CD medication), being 18 or older, and being employed prior to and after diagnosis. The 2-year period was selected to avoid surveying respondents after a significant period since their diagnosis, which could have made it difficult to assess the impact of the disease. To obtain this sample, we collaborated with a survey company that maintains a panel of more than 10,000 people who have recovered from various chronic health conditions. Participants in this panel answer demographic surveys that gather information such as education level, income, and marital status. Through the survey company, we recruited 120 participants. Several steps were taken to reach participants. Initially, our survey company performed initial filtering based on existing profiles. Following the selection process, we contacted potential participants via email or text message to invite them to participate. An explanation of the study was included in this message. Participants who expressed interest in participating in the study were contacted and informed of the study’s goals and the type of survey in more detail. During this conversation, the participants signed consent forms and agreed to a time and place for questionnaire administration. The questionnaires took between 30 and 55 min to complete, and they were conducted in Hebrew or Arabic, Israel’s two main languages. Participants received 100 Ins. (equivalent of $30) as an incentive, and their anonymity was assured.

The demographic characteristics of the participants are described in Table 1. About 58% of participants were men and 42% were women, no significant differences were found between men and women in background characteristics The respondents’ age range was 21–71 years, with an average age of 49.97 (*SD* = 12.53). Additionally, 31.7% reported lower income than average, 31.7% reported average income, and 36.6% reported higher income than average for an Israeli; 68.3% were married; and 31.7% had at least one other employee.

**Table 1:** Background characteristics of the sample (*N* = 120).

Variable	Males (58, 48.3%)	Females (62, 51.7%)	Total sample (120, 100%)
Age, (years)	*M* = 50.28, SD = 11.25 range = 26–69	*M* = 46.19, SD = 14.21 range = 21–70	*M* = 48.17, SD = 12.97, range = 21–70
Education, years	*M* = 14.90, SD = 3.25, range = 10–22	*M* = 15.19, SD = 3.18, range = 10–25	*M* = 15.05, SD = 3.21, range = 10–25
Income			
Below average	16 (27.6%)	22 (35.6%)	38 (31.7%)
Average	18 (31.1%)	20 (32.2%)	38 (31.7%)
Above average	24 (41.3%)	20 (32.2%)	44 (36.6%)
Marital status			
Married or partnered	40 (69%)	42(67.7%)	82 (68.3%)
Non-married	18 (31%)	20 (32.3%)	38 (31.7%)

### Measures

Background details included gender, age, family status, education level, health status, economic status, religiosity, and the effect of COVID-19 on their work.

The work ability limitations of the participants were assessed by a 4-item scale. Participants were asked whether they encountered work related problems during the past month, such as shortened work hours, low productivity, limited activity, or forgoing tasks required for maintaining their businesses such as marketing, because of health conditions. Due to absence of such a tool, the questions were based on literature suggesting work limitations for individuals with chronic health conditions (e.g., Munir et al., 2007). Each item was measured on a 5-point Likert scale ranging from 0 (*not at all*) to 4 (*a great amount*). McDonald’s omega was used to estimate internal consistency. The internal consistency (McDonald’s omega) of this variable was ω = 0.92.

IU was assessed by the Intolerance of Uncertainty Scale, short form (Carleton et al., 2007), validated by Lauriola et al. (2018). Only the 7-item subscale that examines prospective anxiety (fear and anxiety regarding future events) was used. Each item was measured on a 5-point Likert scale (1 = *not at all characteristic of me*, 5 = *entirely characteristic of me*). Example items are: “Unforeseen events upset me greatly” and “It frustrates me not having all the information I need.” A mean score was calculated. High reliability was previously reported (Cronbach’s α = 0.96). The previously translated and validated Hebrew version was used in the present study (Cohen et al., 2022) and translated to Arabic using the back-translation method. The internal consistency (McDonald’s omega) of this variable was (ω = 0.86) Demoralization was measured by the Demoralization Scale II (DS-II). This is the short version of the Demoralization Scale-24—the most widely validated scoring instrument used for assessing demoralization among patients with chronic health conditions (Clarke and Kissane, 2002). The DS-II is a 3-point self-report scale, ranging from 0 (*never*) to 2 (*often*), that features 16 items and two subscales (meaning and purpose, distress and coping ability). Given its revalidation, psychometric strengthening, and simplification, the DS-II is an improved and more practical measure of demoralization for research and clinical use (Robinson et al., 2016a,b). Example statements are: “I do not cope well with life” and “I feel that I cannot help myself.” For clinically significant levels of demoralization, we adopt Bobevski et al. (2022) cutoffs of 10 demoralization symptoms. This scale has been used in previous studies conducted among individuals with CD and was shown to be a reliable measure (*α* = 0.86–0.92; Liao et al., 2017; Wu et al., 2019). The internal consistency (McDonald’s omega) of this variable was (*ω* = 0.92).

Well-being was assessed by the WHO-5 Well-Being Index (Topp et al., 2015). Items include feeling cheerful, active, refreshed, and interested. Each item is rated on a 6-point Likert type scale ranging from 1 (*at no time*) to 6 (*all of the time*), with higher scores indicating greater well-being levels. A mean score was calculated. High reliability was previously reported (Cronbach’s *α* = 0.90). The internal consistency (McDonald’s omega) of this variable was (*ω* = 0.94).

### Data analysis

Descriptive statistics were used to assess frequencies, percentages, means, and standard deviations of demographic and study variables. Pearson correlations between the background and study variables and between study variables were examined. The total, direct, and indirect effects (unstandardized and standardized) were subsequently tested for effect size and statistical significance using a bootstrapping test (5,000 bootstrap samples via PROCESS Model 4) and 95% bias-corrected confidence intervals (CIs) to evaluate the statistical significance of the indirect paths (i.e., work ability limitations to demoralization to well-being). If the interval estimate did not include 0 (Hayes, 2017), the effect was statistically significant. We further applied the Johnson–Neyman technique (Hoyt, 1944) for depicting moderation effects. The Johnson–Neyman technique provides a comprehensive information for reporting how the effect of a predictor on an outcome variable is influenced by the entire range of the moderator. Based on the multiplicative interaction effect model, the Johnson–Neyman technique uses a regression line with the effect of the predictor on the outcome regressed on the moderator to show how the effect changes according to the moderator’s changes. The Johnson–Neyman technique also uses 95% confidence bands around the simple regression line. We performed the Johnson–Neyman analysis to determine how the effect of demoralization on wellbeing varies from being significant or not based on the IU value. Process macro version 3.3 for SPSS (Hayes, 2017) was used. Finally, to test whether this indirect path was contingent on IU levels, we used a PROCESS macro (Model 14) and Johnson– Neyman statistical analysis. Following Regorz’s (2022) recommendations, the model was also tested with the Model 15 program to avoid a possible bias associated with Model 14. Due to almost identical results, confirming no bias, results are presented according to Model 14 (Hayes, 2017).

## Results

The means, standard deviations, and ranges of the study variables are presented in Table 2. Self-employed individuals with CD reported high rates of work ability limitations, high levels of IU, medium levels of demoralization, and medium levels of well-being. The prevalence of demoralization syndrome in our sample was (37.4%); the actual score range was 0.06–1.69, with an average of 0.89 (*SD* = 0.41). Table 2 also shows the correlations between study variables. Work ability limitations was positively associated with IU (*r* = 0.46, *p* < 0.01) and demoralization (*r* = 0.35, *p* < 0.01), but it was negatively associated with well-being (*r* = −0.49, *p* < 0.01). IU and demoralization were also negatively associated with well-being (*r* = −0.56, *p* < 0.01; *r* = −0.26, *p* < 0.01) respectively.

**Table 2:** Means, standard deviations, range, and correlations of the study variables (*N* = 120).

	*M*	*SD*	Theoretical range	Actual range	1	2	3
1. Work ability limitations	2.10	1.30	0–4	0–4			
2. Intolerance of uncertainty	3.40	0.77	1–5	1.57–5.00	0.46*		
3. Demoralization	0.87	0.41	0–2	0.06–1.69	0.35*	0.16	
4. Well-being	2.14	1.20	0–5	0–4	−0.49*	−0.56*	−0.26*

*p < 0.001.

### Mediation and moderation effects

In the first step of the study model assessment, the associations between the outcome variable (well-being) and background characteristics (gender, age, religiosity, education level, health status, economic status, and religiosity) were examined to determine which background variables should be controlled. Only gender was associated with the outcome variables and therefore, we tested separate models for males and females. Since no significant differences were found between the sexes, in the final model gender was controlled.

To assess whether demoralization mediated the association between work ability limitations and levels of well-being, mediation analysis was performed using PROCESS Model 4, the results of which are presented in Table 3. A significant direct association was found between work ability limitations and well-being (*b* = −0.52, *SE* = 0.08, 95% CI [−0.70, −0.35], *p* < 0.001) and demoralization was significantly associated with well-being (*b* = −0.78, *SE* = 0.26, 95% CI [−1.30, −0.26], *p* = 0.004). After controlling for the mediator, the direct effect of work ability limitations was reduced but still significant (*b* = −0.33, *SE* = 0.09, 95% CI [−0.47, −0.29], *p* < 0.001), indicating a partial mediation effect. The indirect effect of demoralization was significant (β = −0.19, *SE* = 0.07, 95% CI [–0.26, −0.12]). Thus, the first hypothesis was confirmed: Work ability limitations were associated with well-being via demoralization; stronger work ability limitations were associated with higher demoralization scores, which were associated with lower well-being scores.

**Table 3:** Mediating effect of IU between physical symptoms and job satisfaction.

Relationship	Total effect	Direct effect	Indirect effect	95% CI	*t*	Conclusion
Work ability limitation → demoralization → well-being	−0.52**	−0.33**	−0.19**	−0.70, −0.35	−6.11	Partial mediation

**p < 0.001.

To examine the second hypothesis, which postulated that IU moderates the relationship between demoralization and well-being, and the third hypothesis, according to which IU moderates the mediating role of demoralization between work ability limitations and well-being, PROCESS Model 14 was performed (see Table 4), controlling for gender and physical symptoms. In accordance with the second hypothesis, the results showed a significant moderating impact of IU on the relationship between demoralization and well-being (*b* = −0.06, *t* = −2.50, *SE* = 0.02, *p* = 0.01). The overall model, including the dependent and interaction variables, was statistically significant [*F*_(5, 114)_ = 17.38, *p* < 0.001]; 43.25% of the variance in well-being was explained by the independent and interaction variables. The interaction added 4% to the explained variance of well-being [Δ*R*^2^ = 0.04, *F*_(1, 114)_ = 5.02, *p* = 0.02]. The results of the Johnson–Neyman analysis showed that the negative impact of demoralization on well being became significant when the IU score was >3.87.

**Table 4:** Conditional effects of demoralization on well-being.

Moderator	Condition	*b*	*SE*	Bootstrap 95% CI	*o*
IU (16th percentiles)	Low	0.34	0.35	−0.35, 1.03	0.33
IU (50th percentiles)	Middle	−0.21	0.22	0.36, −0.65	0.36
IU (84th percentiles)	High	−0.57	0.26	−1.09, −0.48	0.02

**Figure 2:**
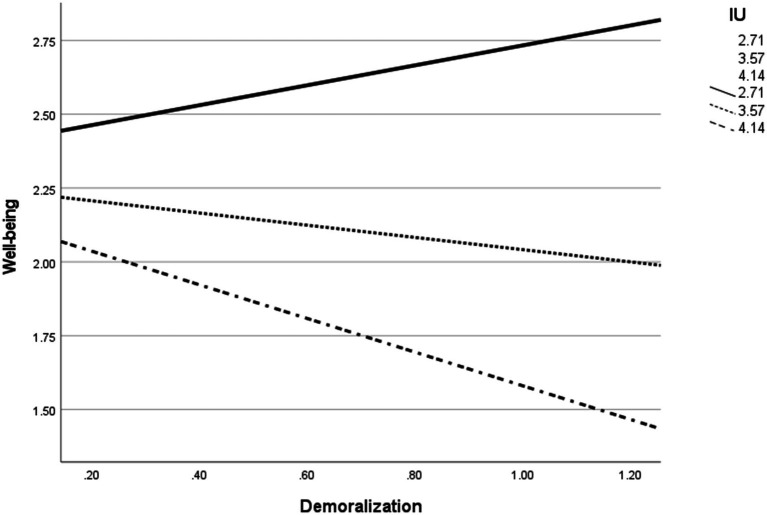
Results of the moderation model of demoralization on well-being in different IU levels.

The graphic representation of the analysis (Figure 2) shows that the slope between demoralization and well-being was steeper at higher levels of IU, compared to low and medium levels of IU. As shown in Table 4, the slope of the association between demoralization and well-being was not statistically significant at low (*b* = 0.34, *SE* = 0.04, *p* = 0.33) and medium (*b* = −0.21, *SE* = 0.22, *p* = 0.36) levels of IU, which means that demoralization was not significantly associated with well-being at low and medium levels of IU. However, with high levels of IU (*b* = −0.57, *SE* = 0.26, *p* = 0.02), the slope for IU was negative and significant. Thus, demoralization was associated with lower well-being levels only among participants with high IU levels.

Finally, the results presented in Table 5 show that in accordance with our third hypothesis, the indirect effect of work ability limitation on well-being through demoralization was contingent on IU levels. The conditional indirect effect was only significant at high levels of IU (*b* = −0.21, 95% CI [−0.25, −0.16]). However, at low (*b* = 0.04, 95% CI [−0.22, 0.29]) and medium (*B* = −0.02, 95% CI [−0.38, 0.00]) levels of IU, the indirect effect was not statistically significant. This indicates that demoralization mediated the relationship between work job limitation and well-being only at high levels of IU.

**Table 5:** Moderated mediation model: indirect effect of work ability limitation through demoralization moderated by IU on well-being.

Moderator	Condition	*b*	*SE*	Bootstrap 95% CI
IU (16th percentiles)	Low	0.04	0.03	−0.22, 0.29
IU (50th percentiles)	Middle	−0.02	0.02	−0.38, 0.003
IU (84th percentiles)	High	−0.21	0.11	−0.25, −0.16

## Discussion

This study extended the existing literature concerning self-employed individuals with CD, providing insight into the impact of IU among CD patients who encounter various levels of uncertainty. Due to their medical situation and need to work independently, they might encounter difficulties in performing their job roles appropriately as self-employed workers, which can affect their health.

The study found that work ability limitations were associated with higher demoralization levels, which were also related to well-being. To clarify, work ability limitations were not only directly associated with well-being but also indirectly associated through demoralization. The well-being levels differed among people with the same level of work ability limitations but different degrees of demoralization. This finding is in accord with previous studies that reported that demoralization boosted the effects of objective stressors on well-being and other outcomes (sleep disturbance) among patients with breast cancer (Hsiu-Ling et al., 2021). The finding that the negative effects of work ability limitations on well-being were amplified as the demoralization level increased indicates that impaired well-being is not a direct and inevitable outcome of work ability limitations but can be amplified when paired with demoralization. Regarding practice, identifying demoralization early in the treatment process with interventions that promote hope and self-control could help patients better cope with serious concerns and uncertainties.

We also found that the prevalence of demoralization syndrome in our sample was (37.8%), higher than the 24–35% prevalence estimated in a recent systematic review of demoralization studies among individuals with progressive disease and cancer (Gan et al., 2022). Measurement factors may have contributed to this high rate because the cutoff for demoralization in this study was a DS-II score ≥ 10 (Bobevski et al., 2022). Other studies have used higher cutoff scores (Bailey et al., 2020; Bobevski et al., 2022). Nevertheless, the rate of demoralization syndrome found in the study is high and warrants attention. In addition, the range of DS-II scores shown in this study was wide, from 1 to 27. A possible explanation for this may be related to the difference in illness severity among participants (from low to high severity) and degree of job requirement complexity (from low to high). The finding that participants with higher demoralization levels reported lower well-being accords with previous findings (Tang et al., 2022; Botto et al., 2023).

A further finding was that the levels of IU determined the extent to which demoralization affected well-being. To clarify, there was no significant association between demoralization and well-being among individuals with low and medium levels of IU; this association was significant only among individuals with high levels of IU. This means individuals with CD and high levels of IU have a higher risk of experiencing impaired well-being. This finding is in accord with previous research that found that mental symptoms are associated with well-being only when IU is high. However, at low levels of IU, this association does not exist (Andrews et al., 2023). This finding is also in accord with Robichaud and Dugas’s (2006) model of IU and demoralization. It seems that demoralized individuals with CD who already feel trapped and helpless (Davey and Wells, 2006) and deal with sources of uncertainty (e.g., related to work) may experience additional worries. These worries are often fueled by IU (Robichaud and Dugas, 2006). Experiencing these repeated and ongoing challenges (e.g., maintaining their business) combined with low ability to tolerate uncertainty may increase feelings of demoralization and exhaustion, which may impair well-being (Butler et al., 1991). In sum, self-employed individuals with different IU levels might be disproportionately affected by their situation. Therefore, it might be helpful to identify individuals with high IU early in the treatment process because they might profit from interventions that foster tolerance of uncertainty and acceptance (Robichaud, 2013).

Situations of uncertainty among CD patients, such as being self-employed, can contribute to demoralization, especially for patients with high IU. As a result, it is important to identify people with CD who are prone to IU. In terms of implications, self-employed individuals with CD should be encouraged to engage in planned treatment programs. Second, it is advisable to provide them with detailed information on their health condition and interventions that promote hope and self-control. Because IU plays a serious role in prolonging worries and uncertainties, interventions that promote tolerance and acceptance are highly recommended in the early stages. In terms of policymaking, it is advisable to provide financial support to self-employed individuals with CD. Several limitations of the study should be underscored. First, we collected all data from a single source, which implies that the data might reflect a single-source bias. Moreover, because this was a cross-sectional study, caution must be exercised regarding the directionality of the relationships among the research variables. Another limitation relates to the inclusion criteria (having been diagnosed with CD in the past 2 years), the survey was administered in relation to the onset of disease, perhaps those who are further out from their disease are more likely to realize that their life has not ended and they can go on and as such have less IU. Nevertheless, to the best of our knowledge, this is the only study involving demoralization and well-being among self-employed individuals with CD. Particularly, it identified how mental states and personality traits were associated with well-being. Further studies are needed that employ longitudinal designs and additional measures of business maintenance to enrich knowledge on optimal rehabilitation processes to support self-employed individuals with CD.

Future studies should include factors that may influence demoralization and qualitatively assess relationships among demoralization, IU, and other factors through in-depth interviews. In addition, researchers should investigate other factors that are influential and significant for individuals with CD, particularly those who encounter other uncertainties beyond their health. Last, it is desirable to further explore the effect of variables such as gender, age, socioeconomic status, and job type on the relationship between work ability limitations and work-related well-being of self-employed people.

In conclusion, the present study explored the understudied population of self-employed individuals with CD. The results demonstrate that a future investigation of this group and related policy activities should consider their mental state (such as demoralization) and personal traits (such as IU) as important factors that affect their well-being. Especially, demoralization as a treatable psychological factor should be emphasized early in therapeutic interventions (Robinson et al., 2015). Also, IU is the fuel that feeds uncertainties and worries to consistently affect individuals; therefore, it is highly recommended to emphasize this issue early in interventions.

## Data availability statement

The raw data supporting the conclusions of this article will be made available by the authors, without undue reservation.

## Ethics statement

Approval was obtained from University of Haifa Committee for Ethical Research with Humans. Consent to participate: informed consent was obtained from all individual participants included in the study. In addition, all methods were performed in accordance with the guidelines and regulations of the University of Haifa Committee for Ethical Research with Humans.

## Author contributions

WS: Conceptualization, Writing – original draft, Writing – review & editing. DK: Conceptualization, Validation, Writing – review & editing.
